# Integrative Machine Learning Approaches for Identifying Loci Associated with Anthracnose Resistance in Strawberry

**DOI:** 10.3390/plants14182889

**Published:** 2025-09-17

**Authors:** Yoon Jeong Jang, Dabin Yun, Wonyoung Shin, Changrim Goo, Chul Min Song, Koeun Han, Seolah Kim, Do-Sun Kim, Seonghee Lee, Youngjae Oh

**Affiliations:** 1 Vegetable Research Division, National Institute of Horticultural and Herbal Science, Rural Development Administration, Jeonju 55365, Republic of Korea; jangyj770@korea.kr (Y.J.J.); hke1221@korea.kr (K.H.); sulah1234@korea.kr (S.K.); greenever@korea.kr (D.-S.K.); 2 Department of Horticulture, Chungbuk National University, Cheongju 28644, Republic of Korea; ydb0889@naver.com (D.Y.); swy@chungbuk.ac.kr (W.S.); hac_0375@chungbuk.ac.kr (C.G.); 3 Department of Agricultural & Rural Engineering, Chungbuk National University, Cheongju 28644, Republic of Korea; song_c_m@chungbuk.ac.kr; 4 Gulf Coast Research and Education Center, Institute of Food and Agricultural Sciences, University of Florida, Wimauma, FL 33598, USA; seonghee105@ufl.edu

**Keywords:** feature selection, genomic prediction, octoploid strawberry, anthracnose resistance

## Abstract

Anthracnose, predominantly caused by *Colletotrichum fructicola*, severely reduces yield in *Fragaria* × *ananassa* production. We assessed ensemble machine learning (ML) frameworks to improve genomic prediction (GP) of resistance using a training population of 300 individuals from six full-sib families. Genotyping with the Axiom^®^ 50K FanaSNP array and phenotyping by AUDPC after artificial inoculation enabled evaluation of five algorithms—G-BLUP, LASSO, LightGBM, Random Forest, and XGBoost—combined with informed feature selection and resampling-based data augmentation (3×, 5×). Ensemble ML models consistently outperformed linear approaches, with Random Forest, LightGBM, and XGBoost achieving the highest accuracies. Marker prioritization revealed that a reduced SNP panel of ~200 markers provided near-maximal predictive performance (R^2^ up to 0.991), demonstrating that compact subsets can support cost-efficient GP. Data augmentation, implemented through the resampling of existing observations rather than the creation of new alleles, improved statistical power and model stability under limited sample sizes. Collectively, this study demonstrates that (i) ensemble ML models deliver superior accuracy for predicting polygenic resistance, (ii) small SNP panels can achieve high efficiency, and (iii) augmentation enhances robustness in resource-constrained breeding populations. These findings establish a scalable and breeder-oriented GP pipeline to accelerate the development of anthracnose-resistant strawberry cultivars.

## 1. Introduction

Strawberries are globally cultivated as economically important and nutritionally valuable berry crops. Their high vitamin C, polyphenol, and antioxidant content contribute to their increasing demand in fresh and processed markets [1]. The cultivated strawberry (*Fragaria* × *ananassa* Duch.) is an allo-octoploid species (2n = 8× = 56) originating approximately three centuries ago via natural hybridization between geographically separated wild octoploid progenitors—*F. chiloensis* native to South America and *F. virginiana* found in North America [2,3]. FAOSTAT (2023) reported that global strawberry production reached approximately 9.56 million tons in 2022, highlighting its economic significance [4]. That year, South Korea ranked ninth among strawberry-producing countries, producing approximately 192,889 tons. Despite its high market value, strawberry cultivation faces increasing challenges owing to climate change and emerging pathogens. Although chemical fungicides are widely used in commercial strawberry production, increasing concerns regarding fungicide resistance, environmental safety, and regulatory constraints highlight the limitations of chemical control.

Among fungal diseases affecting strawberry cultivation, anthracnose—primarily caused by *Colletotrichum* spp.—is highly destructive owing to its broad tissue specificity and severe impact on yield and fruit quality [5,6]. Strawberry anthracnose involves multiple *Colletotrichum* species, primarily from the *C. acutatum*, *C. fructicola* and *C. gloeosporioides* species complexes [7,8]. The causal species vary geographically, with pathogenicity differing according to environmental conditions and plant tissue type (e.g., fruit, petioles, leaves, crowns), leading to substantial yield and quality losses [9]. In North America and Europe, *C. acutatum* is frequently the predominant causal agent, particularly under warm, humid field conditions that favor conidial dissemination and surface infection [6,9,10]. In contrast, *C. fructicola*, previously classified within the *C. gloeosporioides* species complex, has recently emerged as the dominant pathogen associated with anthracnose in East Asia, including South Korea, Japan, and central China [11]. This regional shift in pathogen prevalence highlights the importance of understanding species-specific differences in pathogenicity and host interactions. *C. acutatum* primarily induces visible lesions on fruit, stolons, and petioles under high-moisture open-field conditions. In contrast, *C. fructicola* demonstrates a broader infection profile [12,13], exhibiting strong virulence on foliar tissues and crowns. This can result in systemic colonization and plant collapse, especially under relatively cool and humid conditions common in protected cultivation systems [6,11]. Such aggressive pathogenicity has been confirmed by artificial inoculation studies, where *C. fructicola* caused extensive necrosis on leaves and petioles. In these assays, infected plants show mortality rates of approximately 78% within 30 d post inoculation, highlighting the disease’s severity and cultivar susceptibility under favorable environmental conditions [14].

Advancements in genotyping technologies over the two decades have transformed animal and plant breeding approaches. Genome-wide marker-based prediction methods has enabled the estimation of genetic values in individuals without direct phenotypic evaluation, accelerating breeding timelines [15]. Strawberry breeding is particularly challenging owing to its complex genetic architecture and long breeding cycles, requiring more efficient breeding strategies. Genomic prediction (GP) offers a promising solution by using marker data to predict phenotypic traits and enhance breeding efficiency [16,17]. GP is a quantitative genetics approach that estimates genomic estimated breeding values (GEBVs) by integrating high-density genome-wide marker data with phenotypic information from a training population [17,18]. This framework supports molecular breeding by enabling efficient selection for complex traits without repeated phenotyping, thereby accelerating genetic gain through shorter cycles and improved selection efficiency [19,20,21,22]. Typically, GP models are trained on individuals with both genotype and phenotype data, then applied to predict the genetic merit of candidates with genotype data only. However, the dimensionality imbalance between the number of markers (p) and the number of observations (n)—commonly referred to as the “large p, small n” problem—pose statistical challenges such as overfitting and multicollinearity [23,24].

Genome-wide regression models such as GWAS, G-BLUP, Bayesian, and LASSO have enhanced the predictive accuracy of various traits, including disease resistance, fruit quality, and yield across diverse crops [25,26,27]. These innovations are accelerating genetic gain and supporting long-term sustainability of strawberry production. Recently, Machine Learning (ML) techniques have shown great potential to effectively model complex patterns and capture non-linear relationships within these high-dimensional genomic datasets [28,29]. This growing interest in ML emphasizes the importance of feature selection and understanding model performance trends as feature numbers increase to maximize predictive power while mitigating the challenges posed by the “large p, small n” problem [19,29]. These advancements have been driven by the increasing availability of large-scale, high-dimensional biological data. In plant breeding, ML is increasingly used to predict key agronomic and physiological traits across diverse crop species, including wheat, maize, and strawberry [30,31,32,33]. ML models such as Support Vector Machines (SVMs), Random Forests (RFs), Gradient Boosting Machines (GBMs), and deep learning frameworks such as Convolutional Neural Networks (CNNs) and Recurrent Neural Networks (RNNs), have effectively predicted yield potential, disease resistance, and fruit quality. These predictive models assist breeders in making data-driven selection decisions, accelerating breeding cycles and improving overall crop performance. Furthermore, ML-based approaches help mitigate yield losses caused by environmental variability, promoting more resilient and sustainable agricultural systems.

Therefore, this study aimed to assess the effectiveness of genomic markers for anthracnose resistance in strawberry breeding by evaluating the predictive performance of various GP models across multiple populations. The central hypothesis posited that genomic prediction identifies markers with strong effects on anthracnose resistance, enhancing marker-assisted breeding strategies. To test this, diverse statistical and ML approaches for genomic prediction were implemented. Additionally, the contribution of trait-associated markers in enhancing genomic prediction model accuracy was investigated. The findings of this study provide valuable insights into the genetic architecture of disease resistance and offer practical implications for accelerating anthracnose-resistant strawberry cultivar development via genomic-enabled breeding.

## 2. Results

### 2.1. Model Performance with Increasing Feature Count

**Figure 1:**
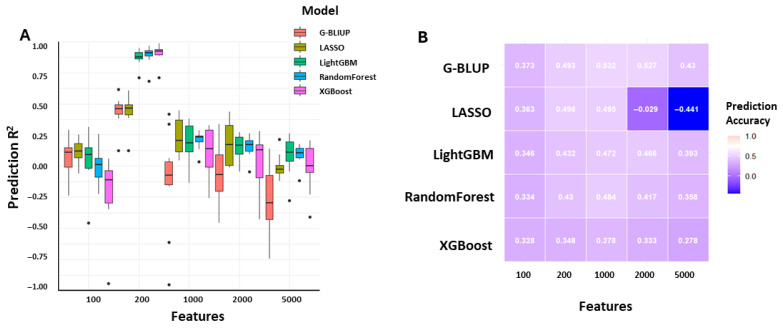
Comparative performance of genomic prediction models across varying marker densities. (**A**) Boxplots show the distribution of cross-validation accuracies for five genomic prediction models—G-BLUP, LASSO, LightGBM, Random Forest, and XGBoost—evaluated with different SNP densities (100, 200, 1000, 2000, and 5000). Accuracies were calculated as Pearson’s correlation coefficients between predicted and observed phenotypes across cross-validation replicates, with statistical differences among models at each marker density assessed using Tukey’s HSD test (*p* < 0.05). (**B**) Heatmap summarizes the corresponding mean prediction accuracies obtained from the independent test set for each model–marker combination, enabling comparison between parametric (G-BLUP and LASSO) and ensemble-based methods (LightGBM, Random Forest, and XGBoost).

To assess how marker set size influences predictive performance of ML models in genomic selection (GS), a feature selection strategy was used to generate five SNP marker subsets of varying dimensionality (100, 200, 1000, 2000, and 5000 markers). Model performance was evaluated using four regression metrics: MSE, RMSE, MAE, and the coefficient of determination (R^2^) (Supplementary Table S1). As illustrated in Figure 1A, cross-validation accuracies varied across models and marker set sizes. In cross-validation (Figure 1A), prediction accuracies were generally low across all models, with substantial variation depending on marker density. Ensemble-based approaches did not consistently outperform parametric methods, and no model exhibited stable superiority under this setting, indicating limited predictive power in cross-validation. Independent test set results (Table 1, Figure 1B), however, revealed a clear advantage for XGBoost. With only 100 SNPs, XGBoost achieved an R^2^ of 0.8724 (RMSE = 2.937), and performance markedly improved at 200 SNPs (R^2^ = 0.9933; RMSE = 0.671). Beyond 200 SNPs, accuracy plateaued, with only marginal gains observed up to 5000 SNPs (R^2^ = 0.9944; RMSE = 0.614). Notably, among parametric models, G-BLUP achieved the highest prediction accuracy at 1000 SNPs (PA = 0.532), whereas LASSO performed the worst at 5000 SNPs (PA = −0.441).

### 2.2. Data Augmentation Drives Performance Gains in Non-Linear Genomic Prediction Algorithms

To overcome the limitations observed in prediction accuracy from the original training dataset, we applied stratified bootstrapping to generate augmented training sets at two levels (3× and 5×). Model performance was assessed across five SNP marker subsets (100, 200, 1000, 2000, and 5000 markers) using five algorithms: G-BLUP, LASSO, LightGBM, Random Forest, and XGBoost. Predictive accuracy was evaluated using four metrics: MSE, RMSE, MAE, and the coefficient of determination (R^2^) (Supplementary Tables S2 and S3).

**Figure 2:**
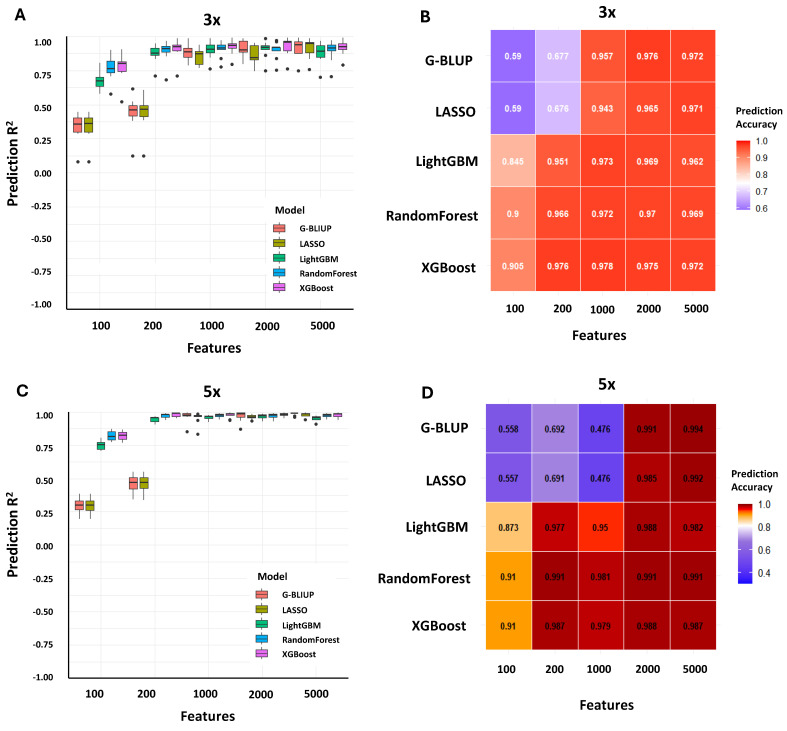
Effect of data augmentation on genomic prediction accuracy across varying marker densities. (**A**,**C**) Boxplots show the distribution of cross-validation accuracies for five genomic prediction models—G-BLUP, LASSO, LightGBM, Random Forest, and XGBoost—evaluated under two augmentation levels, 3× (**A**) and 5× (**C**), with SNP densities of 100, 200, 1000, 2000, and 5000. Accuracies were calculated as Pearson’s correlation coefficients between predicted and observed phenotypes across cross-validation replicates, with statistical differences among models at each marker density assessed using Tukey’s HSD test (*p* < 0.05). (**B**,**D**) Heatmaps summarize the corresponding mean prediction accuracies obtained from the independent test set for each model–marker combination under 3× (**B**) and 5× (**D**) augmentation, enabling comparison between parametric (G-BLUP and LASSO) and ensemble-based methods (LightGBM, Random Forest, and XGBoost).

As shown in Figure 2A,C, cross-validation accuracies markedly improved with data augmentation, particularly for non-linear ensemble models. Ensemble approaches (XGBoost, Random Forest, and LightGBM) consistently outperformed parametric models (G-BLUP and LASSO) and exhibited stable performance across feature dimensions. These patterns were confirmed in Table 2, which highlights XGBoost as the best-performing model under most conditions. For example, at the 3× augmentation level with 200 SNPs, XGBoost achieved an R^2^ of 0.9887 with the lowest associated errors (MSE = 0.778, RMSE = 0.882, MAE = 0.219). At 5× augmentation, further gains were observed, with XGBoost and Random Forest maintaining R^2^ values > 0.99 across SNP subsets of 1000 or more, underscoring the robustness of non-linear algorithms when provided with expanded training data.

Independent test set results, summarized in Figure 2B,D, revealed complementary patterns. At low feature sizes (100 and 200 SNPs), augmentation substantially improved predictive accuracy for ensemble models, with Random Forest and XGBoost reaching the highest accuracies (e.g., PA = 0.91 at 100 SNPs under 5× augmentation). Conversely, parametric models such as G-BLUP and LASSO performed the worst at these low feature counts, with accuracies as low as PA = 0.557 (LASSO, 100 SNPs at 5×). These results indicate that even with relatively small feature sets, non-linear models can achieve strong predictive power under augmented conditions. As the number of SNPs increased to 1000–5000, accuracies across all models converged toward a plateau, with PA values consistently > 0.98 under both 3× and 5× augmentation. These results suggest that beyond a certain marker density, adding more features provides little further benefit to prediction accuracy, a trend that is particularly evident under augmented training conditions.

### 2.3. Impact of Data Augmentation on Model Performance Using a Minimal Set of in Formative Genotypes

**Figure 3:**
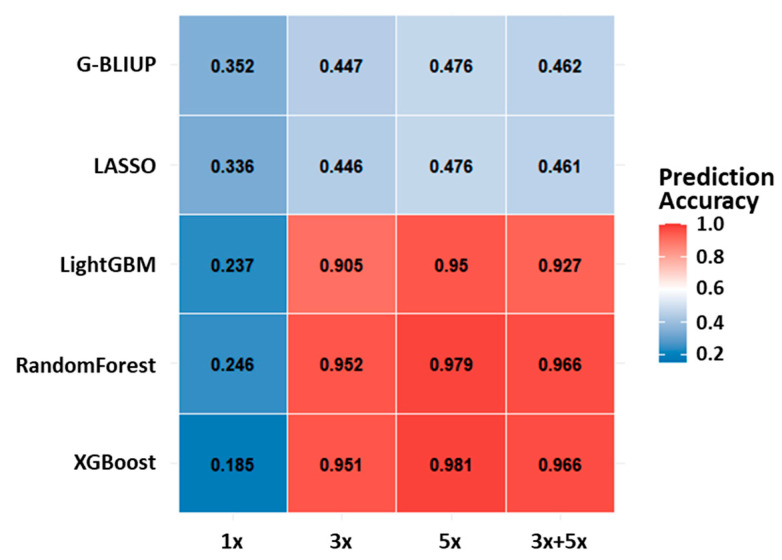
Prediction accuracy of five genomic prediction models using low *p*-value SNP subsets across augmentation regimes. Heatmap showing prediction accuracy for G-BLUP, LASSO, LightGBM, Random Forest, and XGBoost under four augmentation conditions: no augmentation (1×, 30 SNPs), 3-fold (3×, 33 SNPs), 5-fold (5×, 30 SNPs), and combined (3× + 5×, 63 SNPs). Candidate SNPs were selected as common markers across models within each augmentation level based on the lowest *p*-values (Supplementary Table S4) and evaluated on the independent test set. Ensemble models (Random Forest, XGBoost, and LightGBM) achieved substantial accuracy gains with increasing augmentation, whereas linear models (G-BLUP and LASSO) showed only moderate improvements. Color intensity represents prediction accuracy, with red denoting higher and blue denoting lower values.

To evaluate the robustness of SNP subsets identified under different augmentation regimes, we selected common markers across five models (G-BLUP, LASSO, LightGBM, Random Forest, and XGBoost) at each augmentation level (1×, 3×, and 5×) based on the lowest *p*-values (Supplementary Table S4). These markers were then applied to the independent test set (30% of the data held out from training) to assess prediction accuracy, thereby minimizing the risk of circularity between feature selection and model evaluation. Figure 3 presents prediction accuracies obtained using SNP subsets identified at each augmentation level. Ensemble-based models exhibited the most pronounced gains. XGBoost improved from an accuracy of 0.185 with SNPs derived from the 1× condition to 0.951 and 0.981 with 3×- and 5×-derived SNPs, respectively, and retained high accuracy (0.966) when markers from the combined 3× + 5× condition were used. Random Forest and LightGBM also achieved accuracies exceeding 0.90 under both 3× and 5× conditions, whereas a slight decline was observed in the combined set, suggesting redundancy or noise accumulation. Linear models showed more modest responses to augmentation. G-BLUP increased from 0.352 under 1× to 0.476 under 5×, and LASSO from 0.336 to 0.476, but both declined slightly under the combined 3× + 5× condition (0.462 and 0.461, respectively). These results indicate that augmentation-derived SNP panels enhance prediction performance primarily in non-linear ensemble models, while excessive combination of marker sets may reduce efficiency due to information redundancy.

## 3. Discussion

Breeding for resistance to strawberry anthracnose primarily caused by *C. fructicola* is challenging owing to the trait’s quantitative and polygenic nature. Unlike single-gene resistance—where marker-assisted selection (MAS) proves highly effective—*C*. *fructicola* resistance is regulated by numerous minor-effect loci, each contributing incrementally to disease tolerance. This genetic complexity limits the effectiveness of conventional GWAS-MAS pipelines, which are often inadequate to capture the full variation in polygenic traits [11,13,34,35]. To address this limitation, this study employed a GS framework enhanced with ML algorithms to accurately predict breeding values for anthracnose resistance. By integrating data augmentation, feature selection, and ensemble learning models, we constructed a robust prediction pipeline to identify resistance-associated SNPs and select superior genotypes at the early seedling stage. The study prioritized GS coupled with ML rather than GWAS as the core strategy owing to the trait’s biological complexity. Although GWAS effectively detects large-effect QTLs, its locus-by-locus linear framework limits detection of epistasis or small-effect loci, particularly with moderate sample sizes or high-dimensional genomic data [18]. Several studies on complex traits in crops report GWAS’s limited ability to explain a substantial portion of phenotypic variance. In contrast, GS captures genomic-wide variation and estimates GEBVs using both major and minor markers simultaneously [16]. When paired with ML algorithms such as XGBoost, Random Forest, and LightGBM, the GS framework can model nonlinearities, variable interactions, and high-dimensional marker matrices effectively [36].

The complexity of polygenic resistance to fungal pathogens represents a persistent breeding challenge across various crop species. In crops such as wheat (*Triticum aestivum*), maize (*Zea mays*), and rice (*Oryza sativa*), resistance to major fungal diseases—including Fusarium head blight (FHB), northern leaf blight (NLB), and rice blast—is often governed by numerous small-effect loci that conventional GWAS or MAS strategies find difficult to detect [37,38]. For example, wheat’s *Fhb1* QTL aids FHB resistance; however, its effect alone is insufficient to achieve durable resistance owing to the trait’s inherently polygenic nature [39,40]. Similarly, in maize, MAS has failed to effectively pyramid minor alleles for NLB and *Gibberella* ear rot resistance [41]. In rice, MAS also struggles to maintain durable resistance, as major *R*-genes such as *Pi9* and *Pi54* often lose effectiveness under field variability [42,43]. To address these limitations, GS, particularly when integrated with ML algorithms, offers a powerful alternative approach by modeling non-linear genetic interactions. GS captures genome-wide small-effect loci and has shown improved prediction accuracy and selection gain over MAS in various crops. In wheat, GS successfully enhances selection efficiency for FHB resistance beyond the contribution of *Fhb1* alone [37,44]. In maize, GS has demonstrated superior cumulative allele capture for NLB and *Gibberella* ear rot across breeding cycles [38,45,46]. In rice, GS with environment-specific predictive modeling and ML-enhanced feature selection increases selection accuracy for durable, multi-locus resistance [47,48,49]. This paradigm shift toward GS is increasingly relevant to strawberry (*F*. *ananassa*), where resistance to gray mold (*Botrytis cinerea*) and powdery mildew exhibits similar polygenic inheritance patterns. MAS is constrained owing to the scarcity of major-effect loci for these diseases, limiting its utility for long-term resistance breeding [50]. In contrast, GS, especially when augmented with ML techniques, provides a more robust and scalable solution [51,52]. For example, recent studies on strawberry highlight the power of GS for improving complex disease resistance. Research on gray mold (*Botrytis cinerea*) demonstrates GS models using genome-wide SNPs yield moderate to high prediction accuracies (r = 0.41–0.58) for postharvest fruit decay [53]. This approach outperforms single QTL methods, explaining only a small fraction of genetic variance, emphasizing the effectiveness of GS for highly polygenic traits. Consequently, integrating GS with high-throughput phenotyping (HTP) is further advancing the field. For example, when managing powdery mildew, combining GS with canopy reflectance spectrometry enables rapid, non-destructive disease assessment, accelerating selection response and increasing overall genetic gain [54]. Collectively, these cross-crop and strawberry-specific findings highlight the broader applicability of ML-enabled GS frameworks for breeding durable resistance to polygenic fungal diseases in both staple and horticultural crops.

Strawberry anthracnose is a disease complex caused by multiple *Colletotrichum* species, including *C. acutatum*, *C. gloeosporioides*, and *C. fructicola*, each exhibiting distinct pathogenicity profiles [5,9]. Although MAS has been effective against *C. acutatum* using QTL-linked markers, resistance to *C. fructicola* remains unresolved, particularly in Korean breeding populations, owing to its polygenic inheritance [13,35]. Our results confirmed that single-locus selection approaches are inadequate for conferring durable resistance to *C. fructicola*. The relatively small training population of 300 individuals from six full-sib families, while capturing diverse resistance responses, may constrain the generalizability of the models and underscores the need for validation in additional families or independent populations. Nevertheless, the integration of resampling-based augmentation with ensemble machine learning models demonstrated the feasibility of genomic prediction for polygenic resistance. Augmentation increased the effective sample size by resampling existing observations, thereby enhancing statistical power and model stability without introducing novel genetic variants.

For feature selection, we adopted a univariate regression (F-statistics) approach as a pragmatic dimensionality reduction step under the constraints of sample size and population structure. While effective for prioritizing markers with marginal associations, this strategy does not explicitly account for linkage disequilibrium or epistatic interactions, both of which are particularly relevant in the polyploid strawberry genome. To mitigate these limitations, univariate filtering was coupled with ensemble ML models (Random Forest, LightGBM, and XGBoost), which can capture non-linear relationships and partially accommodate LD and epistatic effects during training. Nonetheless, redundancy among selected markers and incomplete representation of non-additive variance remain potential concerns. Recent studies highlight the importance of addressing these complexities: deep learning frameworks have demonstrated the ability to capture dominance and epistatic effects in polyploid crops [55], and multi-kernel genomic prediction models in octoploid strawberry revealed that epistatic variance can account for 16–50% of the total genetic variance, enhancing prediction accuracy compared with additive-only models [56]. These findings underscore the need for future studies to implement LD-aware and epistasis-aware methodologies, particularly as larger and more diverse populations become available.

At the same time, it should be recognized that our cross-validation strategy, conducted at the individual level, may have placed related individuals from the same full-sib or overlapping half-sib families in both training and testing sets. Such relatedness could make prediction easier and lead to somewhat optimistic estimates of accuracy. This caveat is especially relevant when considering the generalization of our models to more divergent germplasm. However, in the context of Korean strawberry breeding, where genetic diversity remains relatively narrow and new populations are largely derived from the same parental combinations, the models developed here remain directly applicable for advancing anthracnose resistance improvement. Looking forward, expanding training resources and applying family-aware validation designs—for example, by assigning entire families to independent validation sets—will provide a stronger basis for assessing model generalizability across broader germplasm pools.

Within this framework, our integrated ML-based SNP selection approach, combining model-derived feature importance with one-way ANOVA filtering, identified 30 robust SNPs associated with anthracnose resistance as measured by AUDPC. This compact SNP subset supports the development of low-density GS panels, maintaining predictive accuracy while reducing genotyping costs. Our approach reflects a broader trend in modern crop genomics, where ML-driven feature selection is increasingly used to extract informative marker subsets from large genomic datasets. Notably, Yeon et al. (2024) demonstrated in tomato that GWAS-derived low-density SNP subsets (192 and 96 SNPs) achieved even higher prediction accuracies for fruit traits than the full SNP panel, underscoring the potential for cost-effective genomic selection [57]. Similarly, our 30-SNP panel represents a functionally enriched set informed by latent patterns learned by ML models and offers a foundation for a low-cost, custom GS platform targeting *C. fructicola* resistance. Beyond strawberry, this ML-guided marker discovery pipeline holds potential for extension to other complex traits and crops characterized by quantitative resistance.

Although cross-validation and consistency checks across models and augmentation regimes were implemented to mitigate the risk of circularity, accuracy estimates may still be slightly optimistic. To address this, we applied augmentation-derived SNP panels to an independent test set, where ensemble machine learning models—particularly XGBoost and Random Forest—achieved marked improvements in predictive accuracy, often exceeding 0.90 under 3× and 5× conditions (Figure 3). In parallel, SNP discovery yielded compact panels of 30–33 markers depending on augmentation level (Supplementary Table S4). Several SNPs were consistently detected across all regimes, representing a robust core subset with stable associations to anthracnose resistance, while augmentation-specific SNPs revealed additional condition-dependent signals. Together, these findings demonstrate that augmentation enhances both predictive performance and marker stability, supporting the development of low-density genotyping panels for cost-effective genomic selection. Independent validation using additional breeding populations, held-out family sets, or permutation-based testing will further confirm the robustness and generalizability of these panels. Interestingly, when markers from both 3× and 5× regimes were combined into a 66-SNP panel, prediction accuracy declined relative to either set alone. This reduction suggests that simply enlarging marker sets by merging augmentation-derived panels may introduce redundancy or noise, offsetting the benefits of augmentation. From a breeding standpoint, these results indicate that smaller, well-defined panels (~30 SNPs) may provide more efficient and reliable prediction than expanded marker sets, while also lowering genotyping costs. Thus, augmentation not only improves the robustness of genomic prediction but also informs the optimal design of parsimonious SNP panels that balance accuracy, stability, and practical applicability in strawberry breeding.

Finally, this study carries a risk of circularity and overfitting, as markers were selected based on their importance across models and then re-evaluated within the same models. Although cross-validation and consistency checks across models and bootstrap conditions were used to mitigate this risk, accuracy estimates may still be slightly optimistic. Independent validation using breeding populations, held-out family sets, or permutation-based significance testing will be essential to confirm the robustness and generalizability of the proposed SNP panel.

## 4. Materials and Methods

### 4.1. Plant Materials and Population Development

Eight breeding parental lines with varying disease response levels, ranging from resistant and moderately resistant to susceptible, were selected to identify genetic factors associated with anthracnose resistance in strawberry. Six full-sib families were developed using eight breeding parents with varying disease responses. The population consisted of 300 individuals, with 50 progeny per family. Because some parents were used in multiple crosses, the design also generated overlapping half-sib relationships among families, providing a structured resource for genomic prediction analyses. Crosses were conducted during the 2022–2023 growing season at the National Institute of Horticultural and Herbal Science (NIHHS). Daughter plants were clonally propagated during the 2023–2024 season in collaboration with the Chungcheongnam-do Strawberry Research Institute.

### 4.2. Genotyping Analysis

Genomic DNA was extracted from seedlings derived from six biparental F_1_ populations developed for anthracnose resistance analysis. Fifty milligrams of young leaf tissue was collected from greenhouse plants and stored at −80 °C in liquid nitrogen until use. Samples were homogenized using a TissueLyser II (Qiagen, Hilden, Germany), and DNA was isolated using a modified cetyltrimethylammonium bromide extraction protocol based on Han et al. (2024) [58]. The extracted DNA was submitted to Thermo Fisher Scientific for genotyping with the Axiom^®^ 50K FanaSNP array (Waltham, MA, USA) [59]. Following quality filtering based on call rate and minor allele frequency (MAF), 27,094 high-quality single nucleotide polymorphism (SNP) markers were retained for downstream analyses. All SNP genotypes were numerically encoded for subsequent statistical analysis. SNP genotyping was performed using Axiom Analysis Suite Software v4.0.3.3, followed by manual quality control. SNP markers were filtered based on a 5% MAF threshold, Hardy–Weinberg equilibrium *p*-value ≤ 1 × 10^−6^, and a 30% missing data rate. Mendelian inconsistencies in parent–offspring trios were identified using PLINK v1.9 [60], and erroneous genotype calls were masked as missing and subsequently imputed. Genotype imputation was performed in PLINK v1.9 using a simple allele-frequency method (major allele imputation), whereby missing genotypes were replaced with the most frequent allele observed within the dataset.

### 4.3. Pathogenicity Assay and Phenotypic Evaluation

Pathogenicity tests were conducted in a plastic greenhouse at the NIHHS using the *C. fructicola* isolate CGF170803. This isolate was cultured on potato dextrose agar (Difco™, BD Diagnostics, Sparks, MD, USA) and incubated at 25 °C for 7 d under continuous light (24 h photoperiod). Conidia were suspended in chilled sterile water and the suspension was filtered through four layers of sterile gauze. The conidial suspension was adjusted to a concentration of 1 × 10^6^ spores/mL using a hemocytometer and was applied to the entire plant via spray inoculation. Each genotype was represented by three clonal plants, which were used as biological replicates for phenotyping in the inoculation assay. Thereafter, plants were covered with plastic film for 48 h to maintain saturated humidity conditions. After 48 h, the film was removed, and disease symptoms were monitored on susceptible genotypes at 8, 15, and 27 days post inoculation (dpi). Disease severity was visually assessed based on the proportion and extent of symptoms observed on petioles and leaves using the following rating scale: 0 = no visible symptoms; 1 = symptoms present on ≤50% of petioles and leaves; 2 = symptoms present on >50% of petioles and leaves; 3 = wilting observed despite soil moisture, with signs of crown tissue necrosis; and 4 = complete plant death. Disease progression over time was quantified using the area under the disease progress curve (AUDPC), calculated from the symptom scores collected at each time point. The resulting AUDPC values were used to evaluate the pathogenicity level of each genotype.

### 4.4. Feature Selection

Feature selection was performed before model training to reduce dimensionality and improve predictive performance and model interpretability [61]. A univariate linear regression-based approach was employed using the SelectKBest class from the Scikit-learn library. The F-statistic (f_regression) was used to evaluate the linear association between each SNP marker and the target variable (AUDPC) [62,63]. For each SNP $X_{i}$, the F-statistic was computed as follows:

$$Fi=\frac{MSRi}{MSEi}=\frac{SSRi/1}{SSEi/(n-2)}$$

where MSRi is the mean-square owing to regression, MSEi is the mean-square error, SSRi is the regression sum of squares, SSEi is the error sum of squares, and n is the number of samples. Features with the lowest *p*-values associated with these F-statistics were retained, and five different feature sets comprising the top 100, 200, 1000, 2000, and 5000 SNPs were constructed for comparative analysis. The same feature selection procedure was uniformly applied to all datasets to ensure consistency across experiments. The selected SNP subsets were also used without modification in downstream analyses involving augmented datasets. All features were standardized using Z-score normalization before model fitting to eliminate the effect of scale differences. For each feature x, the standardized value z was computed as:

$$z\;=\;\frac{x-\mu \;}{\sigma }$$

where μ and σ represent the mean and standard deviation of the feature, respectively. This transformation ensures that each predictor has a mean of zero and a standard deviation of one, beneficial for model convergence and comparability.

### 4.5. Data Preprocessing and Augmentation

To enhance the predictive robustness of genotype-to-phenotype modeling in this study, a multi-step data transformation pipeline grounded in biological interpretability and statistical rigor was implemented. Specifically, the continuous AUDPC trait was discretized into three ordinal resistance categories based on the empirical distribution of the data: resistant (AUDPC 16.0–32.5), moderately resistant (32.8–43.6), and susceptible (44.0–60.6). Classification thresholds were placed near the first (Q1) and third (Q3) quartiles to ensure balanced sample sizes across categories. Since no standardized biological thresholds exist for AUDPC-based resistance classification in strawberry, adopting a data-driven, reproducible framework allowed rational categorization. This discretization enabled balanced representation across resistance classes and facilitated downstream classification analysis. Following feature selection, the dataset—comprising individual F_1_ identifiers, selected SNP features, and associated AUDPC values—underwent additional preprocessing to ensure data integrity. Outlier detection was performed using Z-score normalization, with samples exceeding |Z| > 4 excluded to minimize noise and non-biological variance. Of the initial 300 individuals, two (approximately 0.7%) were excluded based on the |Z| > 4 criterion, resulting in a final dataset of 298 individuals for all subsequent analyses. Z-score was computed using the zscore() function from the SciPy library [64]. Subsequently, the dataset was partitioned into training and testing subsets in a 70:30 ratio using the train_test_split() function from the Scikit-learn package, with a fixed random seed (random_state = 42) to ensure replicability [62]. A stratified bootstrap augmentation strategy was used to mitigate potential class imbalance and improve model generalization. Training sets were synthetically expanded to 3- and 5-fold their original sizes using the resample() function, with stratification to preserve the phenotypic distribution. To rigorously prevent information leakage, augmented data were applied exclusively to the training sets and were never included in validation folds or the independent test set. During cross-validation, augmentation was likewise performed only on the training portion of each fold, while the corresponding validation folds consisted solely of unaugmented data, ensuring that augmented samples did not enter the validation or testing process. This augmentation process maintained original class proportions while increasing sample diversity, improving model robustness [65]. These preprocessing and augmentation procedures provided a biologically grounded framework for evaluating model performance across varying feature dimensions (100, 200, 1000, 2000, and 5000 SNPs) and data volumes (original, 3×, and 5× augmented sets), supporting robust phenotypic prediction in plant molecular breeding.

### 4.6. Genomic Selection Model Training and Evaluation

To evaluate predictive performance under different feature dimensionalities and data augmentation conditions, five widely used machine learning (ML) regression algorithms were implemented: Forest, Extreme Gradient Boosting (XGBoost), Light Gradient Boosting Machine (LightGBM), Least Absolute Shrinkage and Selection Operator (LASSO), and Ridge Regression. Notably, Ridge Regression is mathematically equivalent to the Genomic Best Linear Unbiased Prediction (G-BLUP) framework, which is widely applied in genomic selection [66]. The hyperparameters were configured as follows: for Random Forest, n_estimators = 100 and random_state = 42 were used; for XGBoost, objective = “reg:squarederror”, n_estimators = 100, and random_state = 42 were applied; and for LightGBM, n_estimators = 100 and random_state = 42 were used. For LASSO, the optimal α was selected via five-fold cross-validation (cv = 5), while for Ridge (G-BLUP), α was chosen through cross-validation from the candidate set {0.1, 1.0, 10.0}. All other hyperparameters not specified were left at their respective library defaults. Each model was trained on datasets with varying SNP feature counts (100, 200, 1000, 2000, and 5000) and three data volume conditions (original, 3×, and 5× augmented sets). To assess model generalizability, 10-fold cross-validation was used by randomly partitioning the dataset into 10 equal-sized subsets (folds), with each fold used once as a validation set and the remaining nine as the training set. This process was repeated 10 times, and average performance metrics—coefficient of determination (*R*^2^), mean-squared error (MSE), root mean-squared error (RMSE), and mean absolute error (MAE)—were calculated to evaluate model robustness. Final predictive accuracy was assessed using an independent test set comprising 30% of the data.

### 4.7. Comparative Evaluation of Predictive Models Using Resistance-Associated SNP Subsets

Based on the results from varying data augmentation levels (1×, 3×, 5×) and feature counts (100, 200, 1000, 2000, and 5000), a set of SNP markers significantly associated with anthracnose resistance was identified. Resistance-associated SNPs were selected by integrating markers that consistently ranked among the lowest *p*-values across the five ML models (G-BLUP, LASSO, LightGBM, Random Forest, and XGBoost) within the training set under each augmentation regime. Accordingly, three distinct SNP subsets were constructed: the 1× subset (30 SNPs), 3× subset (33 SNPs), and 5× subset (30 SNPs), together with an additional combined subset (3× + 5×, 66 non-redundant SNPs). Each subset was subsequently used as predictor variables to retrain and evaluate the five ML models. To ensure clarity, SNP selection was conducted exclusively within the 70% training set, while the remaining 30% test set was reserved for independent validation. Within the training set, 10-fold cross-validation was performed to optimize model parameters, but no augmented data were applied during this phase to isolate the effect of marker selection on predictive performance. Model performance was evaluated using multiple statistical metrics, including R^2^, MSE, and MAE. Reliability was further assessed through scatter plots of observed versus predicted AUDPC values and residual distribution analysis. To provide an additional measure of predictive ability, GEBVs were calculated using leave-one-out cross-validation, and Pearson’s correlation coefficients between predicted GEBVs and observed AUDPC values were computed.

## 5. Conclusions

A key contribution of this study is the demonstration that a small, optimized SNP set can deliver high prediction accuracy, enabling cost-effective genomic prediction (GP) in resource-limited breeding programs. Data augmentation (3× and 5×) enhanced model stability under limited sample sizes, a common constraint in horticultural breeding populations. This approach mitigates overfitting and improves generalization by diversifying training data—a strategy transferable to other trait contexts. Collectively, our findings present a scalable, biologically informed, and economically viable pipeline for early-stage GS targeting quantitative disease resistance. By overcoming GWAS limitations and using ensemble ML models, the proposed strategy shortens breeding cycle, reduces phenotyping costs, and accelerates the development of anthracnose-resistant strawberry cultivars. Moreover, the data-driven marker prioritization strategy and family-aware validation framework proposed in this study provide a reproducible model that can be extended to other polygenic disease resistance traits and horticultural crops. The reduced SNP panel offers a practical and affordable tool for integration into breeding pipelines, supporting faster genetic gain and precision breeding. Future research incorporating independent validation populations and haplotype-based analyses will further strengthen the robustness and translational potential of this approach.

## Figures and Tables

**Table 1:** Independent validation results showing the best-performing model for each SNP panel size. Values represent the highest-performing model for each SNP subset size in the independent test set.

Number of Features	Best Model	R^2 a^	RMSE ^b^	MSE ^c^	MAE ^d^
100	XGBoost	0.8724	2.937	8.626	1.29
200	XGBoost	0.9933	0.671	0.45	0.141
1000	XGBoost	0.9944	0.614	0.377	0.091
2000	XGBoost	0.9944	0.614	0.377	0.091
5000	XGBoost	0.9944	0.614	0.377	0.091

^a^ coefficient of determination; ^b^ root mean square error; ^c^ mean square error; ^d^ mean absolute error.

**Table 2:** Performance of the best genomic prediction models across SNP densities and augmentation levels in the independent test set.

Features	Augmentation	Best Model	R^2 a^	RMSE ^b^	MSE ^c^	MAE ^d^
100	3×	XGBoost	0.8824	2.8403	8.0673	1.3259
100	5×	Random Forest	0.8586	3.2169	10.3487	1.369
200	3×	XGBoost	0.9887	0.8821	0.7781	0.2187
200	5×	XGBoost	0.9918	0.774	0.599	0.1634
1000	3×	XGBoost	0.9914	0.7664	0.5874	0.1313
1000	5×	XGBoost	0.9936	0.6818	0.4649	0.102
2000	3×	XGBoost	0.991	0.766	0.588	0.131
2000	5×	G-BLUP	0.9937	0.6812	0.4641	0.1294
5000	3×	G-BLUP	0.992	0.765	0.585	0.17
5000	5×	G-BLUP	0.9937	0.6812	0.4641	0.1294

Model performance was evaluated using four metrics: ^a^ coefficient of determination; ^b^ root mean square error; ^c^ mean square error; ^d^ mean absolute error.

## Data Availability

Genotype data have been deposited in the National Agricultural Biotechnology Information Center (https://nabic.rda.go.kr, accession number NV-0934, NV-0935, NV-0936 and NV-0937, accessed on 2 December 2024).

## Supplementary Materials

The following supporting information can be downloaded at: https://www.mdpi.com/article/10.3390/plants14182889/s1; Supplementary Table S1: Predictive performance of five genomic selection models across varying SNP feature subset sizes; Supplementary Table S2. Predictive performance of five genomic prediction models under 3× data augmentation across five SNP marker subsets; Supplementary Table S3. Predictive performance of five genomic prediction models under 5× data augmentation across five SNP marker subsets; Supplementary Table S4. Consistently selected SNP markers across different data augmentation levels.

## Abbreviations

The following abbreviations are used in this manuscript:

CNN	convolutional neural network
FAOSTAT	Food and Agriculture Organization Corporate Statistical Database
G-BLUP	genomic best linear unbiased prediction
GBM	gradient boosting machine
GEBV	genomic estimated breeding value
GP	genomic prediction
GWAS	genome-wide association study
LASSO	least absolute shrinkage and selection operator
ML	machine learning
RF	random forest
RNN	recurrent neural network
SNP	single-nucleotide polymorphism
AUDPC	area under the disease progress curve
SVM	support vector machine
